# Recombinant Human MFG-E8 Attenuates Intestinal Injury and Mortality in Severe Whole Body Irradiation in Rats

**DOI:** 10.1371/journal.pone.0046540

**Published:** 2012-10-08

**Authors:** Michael A. Ajakaiye, Asha Jacob, Rongqian Wu, Weng Lang Yang, Jeffrey Nicastro, Gene F. Coppa, Ping Wang

**Affiliations:** Center for Immunology and Inflammation, The Feinstein Institute for Medical Research, and Department of Surgery, Hofstra North Shore-LIJ School of Medicine, Manhasset, New York, United States of America; National Cancer Institute, United States of America

## Abstract

The gastrointestinal (GI) syndrome component of acute radiation syndrome (ARS) results from depletion of immature parenchymal stem cells after high dose irradiation and contributes significantly to early mortality. It is associated with severe, irreparable damage in the GI tract and extremely low survival. There is a need for the development of viable mitigators of whole body irradiation (WBI) due to the possibility of unexpected high level radiation exposure from nuclear accidents or attacks. We therefore examined the effect of recombinant human milk fat globule-EGF factor 8 (rhMFG-E8) in mitigating damage after WBI. Male Sprague-Dawley rats were exposed to 10 Gy WBI using Cesium-137 as the radiation source. The animals in the treatment group received rhMFG-E8 (166 µg/kg BW) subcutaneously once a day with the first dose given 6 h after WBI. Blood and tissue samples from the ileum were collected after 3 days of treatment. A separate cohort of animals was treated for 7 days and the 21 day mortality rate was determined. Treatment with rhMFG-E8 significantly improved the survival from 31% to 75% over 21 days. Furthermore, rhMFG-E8 treatment resulted in a 36% reduction in the radiation injury intestinal mucosal damage score, corresponding to visible histological changes. MFG-E8 gene expression was significantly decreased in WBI-induced animals as compared to sham controls. Treatment with rhMFG-E8 increased p53 and p21 expression by 207% and 84% compared to untreated controls. This was accompanied by an 80% increase in the expression of anti-apoptotic cell regulator Bcl-2. p53 and p21 levels correlate with improved survival after radiation injury. These cell regulators arrest the cell after DNA damage and enable DNA repair as well as optimize cell survival. Taken together, these results indicate that rhMFG-E8 ameliorates the GI syndrome and improves survival after WBI by minimizing intestinal cell damage and optimizing recovery.

## Introduction

The current widespread use of radioactive materials has resulted in the realization of the serious and dangerous effects of radiation exposure. As evidenced by the Chernobyl nuclear disaster of 1986 and more recently with the massive radiation leak at the Fukushima I Power plant, massive unforeseen radiation exposure is a possibility which we must plan for and mitigate. This is further necessitated by the risk of nuclear warfare or the utilization of a dirty bomb by terrorists. Major strides have been made in minimizing the effects of planned radiation exposure, especially in radiology and radiotherapy. Radio-protectors have been developed which have shown efficacy in animal and human studies, and one of these radio-protectors, amifostine is already in clinical use [1]–[3]. However, amifostine is limited by its route of administration and toxicity which would minimize its usefulness in the event of an imminent nuclear disaster. Therefore, there has been an unmet need in the development of effective mitigators of radioactive damage.

Acute radiation syndrome (ARS) is an acute illness caused by rapid exposure of most or all of the body to a high dose of penetrating radiation. Its major cause is the depletion of immature parenchymal stem cells in specific tissues. The gastrointestinal (GI) syndrome, one of the three classic ARS syndromes contributes significantly to early mortality and several debilitating complications that follow severe acute radiation exposure. Occurrence of the GI syndrome is associated with extremely low survival: destructive and irreparable changes occur in the GI tract with loss of intestinal crypts and breakdown of the mucosal barrier. At higher radiation doses, the mortality rate of the gastrointestinal syndrome exceeds that of the hematopoietic syndrome with most victims dying within 2 weeks [4], [5].

Milk fat globule-EGF factor 8 (MFG-E8) is a secreted integrin-binding glycoprotein which was first identified as one of the major proteins associated with the milk fat globule membrane in the mouse mammary epithelium [6]. MFG-E8 is widely expressed in different species [7], [8]. The human homolog contains 387 amino acids and has been identified by several other names including Lactadherin, SED1 and BA46. MFG-E8 consists of two-repeated EGF-like domains, a mucin-like domain, and two-repeated discoidin-like domains (C-domains); it contains an integrin-binding motif (RGD sequence) and is reported to have two splice variants. A longer splice variant is expressed in a lactation-dependent manner in mammary tissues while the shorter splice variant is expressed ubiquitously in many tissues. MFG-E8 is a potent opsonin for the clearance of apoptotic cells. It is produced by mononuclear cells of immune-competent organs including the spleen and the liver. MFG-E8 is known to participate in a wide variety of cellular interactions, including phagocytosis of apoptotic cells, adhesion between sperm and the egg coat, repair of intestinal mucosa, mammary gland branching morphogenesis and angiogenesis [8]–[11].

Increasing danger of nuclear attacks, accidents and potential terrorism has caused major concern towards radiation exposure and development of therapies for radiation mitigation is of significant value. Gastrointestinal injuries due to radiation exposure cause high mortality and intestinal crypt cells are extremely sensitive to radiation. Cell proliferation, differentiation, and migration are crucial events required for the maintenance of an intact epithelial layer. MFG-E8 plays an important role in the maintenance of intestinal epithelial homeostasis and the promotion of mucosal healing [7], [12]–[14] which are essential attributes in mitigation of GI impairment after ionizing radiation. Therefore, in the present study, we examined the effect of recombinant human MFG-E8 (rhMFG-E8) in mortality and intestinal damage after exposure to high dose ionizing radiation (10 Gy) in male Sprague-Dawley rats.

## Materials and Methods

### Experimental animals

Male Sprague-Dawley rats (250–350 g) purchased from Charles River Laboratories (Wilmington, MA, USA) were used. The rats were housed in a temperature-controlled room on a 12-h light/dark cycle and fed on a standard Purina rat chow diet. Animal experimentation was carried out in accordance with the Guide for the Care and Use of Laboratory Animals. This project was approved by the Institutional Animal Care and Use Committee (IACUC) of the Feinstein Institute for Medical Research.

### Animal model of whole body irradiation

Rats were exposed to whole body irradiation (WBI) of either 7.5 or 10 Gray (Gy) using a Gammacell® 1000 Irradiator (Atomic Energy of Canada Ltd) [radiation source: Cesium-137 (^137^Cs)]. The animals were sedated with intra-peritoneal pentobarbital (40 mg/kg BW) prior to irradiation. During radiation, the container rotated continuously in front of the radiation source for even exposure. The animals were then returned to their cages, and food and water were provided. The lethal irradiation dose for 70% of the animals at 21 days (LD_70_/21) was initially determined to be 10 Gy delivered at a dose rate of approximately 2.5 Gy/min for 4 min. Subsequent experiments were performed at a total radiation dose of 10 Gy.

### Preparation and administration of rhMFG-E8

Human MGF-E8 is a 387 amino acid (aa) precursor that contains a 23 aa signal sequence and a 364 aa mature region (SwissProt # Q08431) was synthesized by our laboratory. The recombinant protein was greater than 99% pure, identified as human MFG-E8 with 95% confidence, and was rendered endotoxin free with Triton-X-114 treatment [15]. Rats were exposed to WBI as described above and randomly assigned to sham, treatment or vehicle groups. Animals in the treatment group received rhMFG-E8 (166 µg/kg BW) subcutaneously once a day with the first dose given 6 h after WBI. The animals received a total of 3 doses and were sacrificed 18 h after the last dose (or 72 h after WBI). In the Vehicle group, rhMFG-E8 was replaced with an equivalent volume of normal saline. All other parameters remained unchanged. Age and weight matched non-irradiated animals were used as sham-irradiated controls.

### Survival study

To assess the survival benefits of rhMFG-E8, additional groups of animals were exposed to 10 Gy WBI and treated with rhMFG-E8 (166 µg/kg BW) subcutaneously once a day with the first dose given 6 h after WBI for 7days and observed for 21 days and the survival was recorded. The surviving animals beyond 21 days were then euthanized.

### Histopathology

Samples of the ileum from Sham, Vehicle and treatment groups from the 72 h time point were harvested 5 mm and 20 mm from the ileo-cecal junction. Four 2 mm sections from each animal were fixed in 1∶ 10 buffered formalin and embedded in paraffin. Tissue blocks were sectioned at a thickness of 5 mm, transferred to glass slides, and stained with hematoxylin/eosin. The slides were examined with a Nikon Eclipse Ti inverted microscope, and intestinal injury was analyzed. We developed a seven point scoring system, the radiation injury intestinal mucosal damage score (RIIMS, Range 7–32, Table 1) assessing changes in villus morphology, height and cell type composition, crypt cellular and nuclei appearance, lymph congestion and mucosal necrosis and exfoliation to grade the severity of damage. Computerized morphometric measurements were made with NIS-Elements BR laboratory image analysis system software: Villus length and crypt depth was measured in alternate villi using in 3–4 histological sections from each animal and measured. The number of enterocytes and goblet cells in neighboring villi were then counted under high magnification. Forty villi from 4 different parts of each sample slide were sequentially chosen and the average counts were utilized. Histology of the ileal tissue from 4 different animals were analyzed in each group.

**Table 1:** The radiation injury intestinal mucosal damage score (RIIMS).

**A**	**Increase in goblet cells**	D	**Crypt cellularity/regeneration**
1	No increase in goblet cell number	1	Normal crypt cellularity
	(defined by goblet cell/enterocyte ratio)	2	Mild hypo/hypercellularity
	(+/−10% of sham average)	3	Marked reparative/inflammatory changes
2	>10–25% increase or >10%–100% decrease	4	Abnormal crypt regeneration/Marked
3	>25–50% increase or >100% decrease		crypt hypocellularity
4	>50–100% increase	E	**Crypt nuclei appearance**
5	>100–200% increase	1	Normal nuclear appearance
6	>200% increase	2	Mild nuclear atypia/increased mitosis
**B**	**Villus Length: Villus length as a percentage**	3	Moderate nuclei atypia
	**of normal (sham)**	4	Severe nuclear abnormalities
1	Normal length (−5 to +10%)	F	**Lymph congestion**
2	5–10% shortening or ≥10% increase in length	1	No central villus lymph vessel congestion
3	>10–20% shortening	2	Mild
4	>20–30% shortening	3	Moderate
5	>30–40% shortening	4	Severe
6	>40% shortening	G	**Mucosal Necrosis/exfoliation**
C	**Villus shape/morphology**	1	Normal brush border
1	Normal morphology	2	Mild exfoliation brush border
2	Mild abnormalities	3	Loss of brush border with mild exfoliation
3	Forked, fused villi	4	Superficial ulcers
4	Flattening, loss of finger-like projections		

RIIMS is a seven point scoring assessing changes in villus morphology, height and cell type composition, crypt cellular and nuclei appearance, lymph congestion and mucosal necrosis and exfoliation to grade the severity of damage. Scores range from 1–6. The minimum collated score of 7 corresponds to normal mucosa with a maximum score of 32 indicating the worst possible damage.

### Western immunoblotting

Ileal tissue lysates (80–100 µg) were electrophoresed on NuPAGE 4–12% Bis-Tris gels and transferred to 0.2 µm nitrocellulose membrane (Invitrogen, Carlsbad, CA). The membranes were blocked in TBS-T (10 mM Tris-HCl, 150 mM NaCl, 0.1% Tween-20) containing 5% non-fat milk for 1 h at room temperature. Western blotting was performed using the following primary antibodies: rabbit anti-p21 antibody (C-19) and rabbit anti-Bcl-2 polyclonal antibody (N-19) (1∶1000) (Santa Cruz Biotechnology). After incubation of the primary antibodies overnight at 4°C, the membranes were washed with TBS-T. Immunoreactive bands were detected using HRP-linked anti-rabbit IgG (1∶10,000) (Southern Biotech, Birmingham, AL) and the Enhanced Chemiluminescence (ECL) Western blot detection kit (Amersham, Piscataway, NJ). The immunoblots were exposed to X-ray film and analyzed with the NIH ImageJ analysis system. Mouse anti-ß-actin monoclonal antibody (1∶20,000) (Sigma) was used as a loading control in all Western blot experiments.

### Total RNA extraction and real time PCR

Total RNA was extracted from the ileum by Tri-Reagent (Molecular Research Center, Cincinnati, OH). RNA (5 µg) from each sample was reverse-transcribed in a 20 µl reaction volume containing 50 mM KCl, 10 mM Tris-HCl, 5 mM MgCl_2_, 1 mM dNTP, 20 U RNase inhibitor, 2.5 mM oligod (T)_16_ primer, and 50 U reverse transcription. The reverse transcription reaction solution was incubated at 42°C for 1 hour, followed by heating at 95°C for 5 minutes; 1 µl cDNA was amplified with 0.15 µM each of 3′ and 5′ primers specific for rat p53 and p21. Rat glyceraldehyde 3-phosphate dehydrogenase (G3PDH) was used as the housekeeping gene. The primers are as follows: 5′- TGA GGA ACA AGG AAC CAG -3′ (forward) and 5′ –GGA AGG ACA CGC ACA TAG -3′ (reverse) for MFG-E8, 5′- CCC CAC CGC CTG TAA GAT T -3′ (forward) and 5′- ATG GGT CCG GAG GAT ACA GAT -3′ (reverse) for p53 (NM_030989), 5′- CGG GAC CGG GAC ATC TC -3′ (forward) and 5′- CGG CGC TTG GAG TGA TAG AA -3′ (reverse) for p21 (U24174), and 5′-TGA AGG TCG GTG TCA ACG GAT TTG GC-3′ (forward) and 5′-CAT GTA GGC CAT GAG GTC CAC CAC-3′ (reverse) for G3PDH (M17701). Each cycle consisted of 30 seconds at 94°C, 30 seconds at 60°C, and 45 seconds at 72°C.

### Irradiation of intestinal epithelial cells (IEC-6) and MFG-E8 treatment

Rat small intestinal cell line, IEC-6 cells were obtained from American Type Culture Collection (ATCC), were cultured in DMEM media (Invitrogen) with 10% FBS, penicillin and streptomycin, and kept in 37°C incubator under humidified condition containing 5% CO_2_. Cells were plated in 96-well plates overnight and irradiated at 8 Gy using an X-ray irradiator (RS-2000 Biological Irradiator, Rad Source). In additional aliquot of cells, they were treated with rhMFG-E8 (0.5 µg/ml) 1 h prior to and immediately after irradiation, and stained with crystal violet at 48 h after treatment.

### Statistical analysis

All data are expressed as mean ± SE and analyzed by one way analysis of variance (ANOVA) and compared using Student Newman Keuls test. The survival curves were plotted using the Kaplan-Meier Analysis and the curves were subjected to the Log Rank test. The differences in values were considered significant if p<0.05.

## Results

### rhMFG-E8 improves survival after whole body irradiation (WBI)

**Figure 1:**
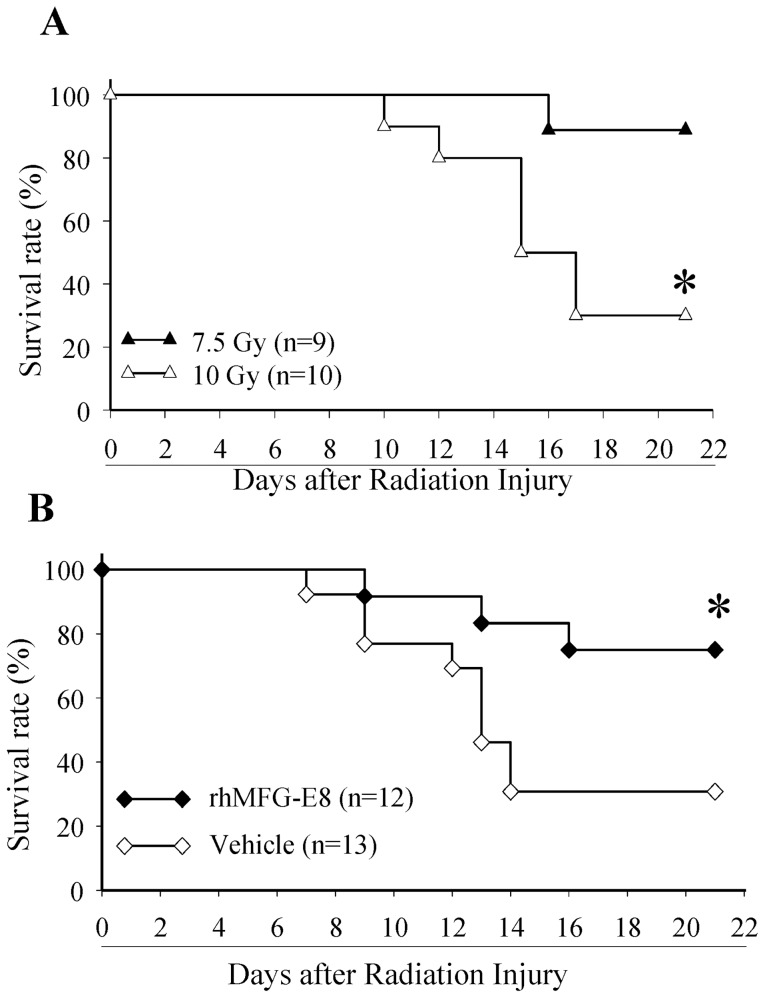
LD_70_/21 and rhMFG-E8 survival curves. (A). Male Sprague-Dawley rats were subjected to WBI using 7.5 and 10 Gy, and observed for 21 days. (B). Rats underwent WBI using 10 Gy, treated with rhMFG-E8 or Vehicle and observed for 21 days. The survival rate was estimated by the Kaplan-Meier method and compared by Log Rank test. * P<0.05 vs. 7.5 Gy (Fig. 1A) or Vehicle (Fig. 1B).

High dose WBI is associated with high mortality. We determined the LD_70_/21 of acute WBI for our experimental cohort (healthy adult male Sprague-Dawley rats) to be 10 Gy (Figure 1A). To determine the beneficial effects of rhMFG-E8, we administered rhMFG-E8 (166 µg/kg BW) subcutaneously once a day for 7 days. The first dose was given 6 h after WBI. Animals were allowed standard Purina chow and water *ad libitum* and observed over 21 days. The mortality rate was compared to that of WBI rats treated with equivalent volumes of normal saline given subcutaneously. Treatment with rhMFGE-8 dramatically improved the survival in WBI rats from 31% to 75% (Figure 1B). In addition to recording the mortality rate, the body weight of the animals was assessed for 21 days. Interestingly, both vehicle and MFG-E8 treated animals that died prior to 21 days had lower body weight than their pre-treatment weight. On the other hand, both vehicle and MFG-E8 treated groups of animals that survived for 21 days restored their body weight to their pre-treatment weight. This indicated a strong correlation between body weight change and survival after WBI. However, none of the animals gained any significant amount of body weight from their pre-treatment weight for the experimental period of 21 days. In a separate cohort, after irradiation and treatment, the animals were kept in metabolic cages. The food intake, stool weight, water intake and urine output were measured for 7 days. The results showed that average stool weight was lower in the MFG-E8 treated rats as compared to vehicle group which was indicative of diarrhea or loose stool in the vehicle group as opposed to normal stool in the MFG-E8 treated rats. No major changes were observed in all other parameters studied.

### rhMFG-E8 preserves intestinal structure and function after WBI

**Figure 2:**
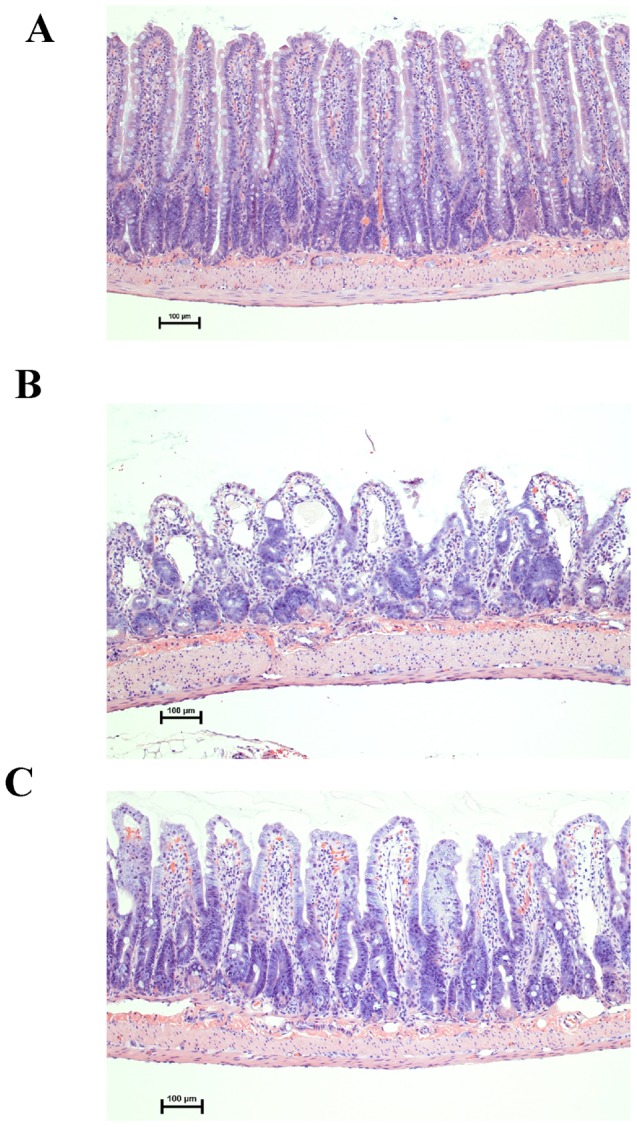
Histology of rat ileum 72 h after WBI. Histological sections of the rat ileum from non-irradiated (sham) animals (A), Vehicle (B) and rhMFG-E8 treated animals (C) harvested 72 h after WBI (×20 magnification).

**Figure 3:**
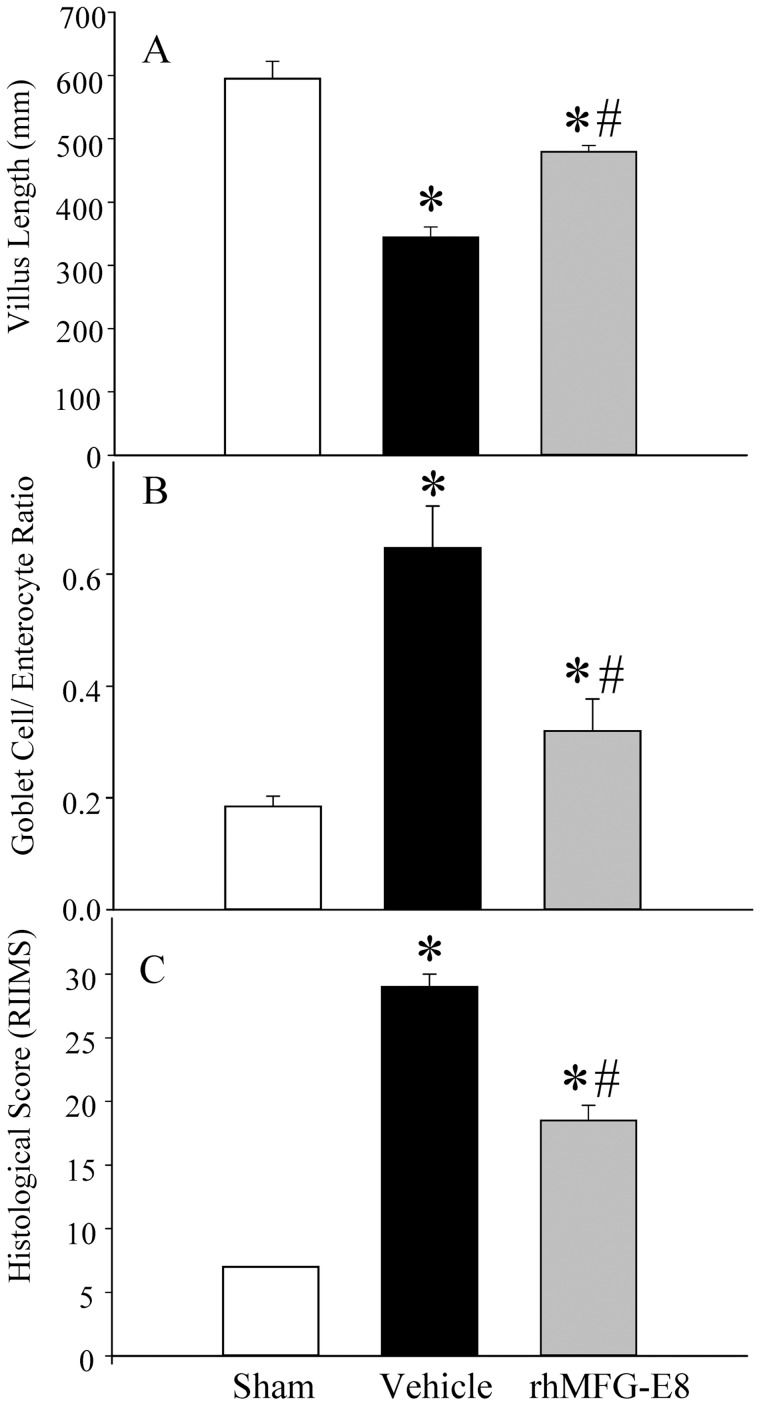
Morphometric histological measurements. Computerized morphometric measurements were made with NIS-Elements BR laboratory image analysis system software. Comparison of mean villus length (A), Goblet cell/enterocyte ratio (B) and the radiation injury intestinal mucosal damage score (RIIMS) were analyzed. Data are presented as mean ± SE (n = 4) and compared with Student Neuman Keuls test by ANOVA. * P<0.05 vs. Sham; # P<0.05 vs. Vehicle.

To determine the effect of WBI on gut morphology and function, we examined hematoxylin and eosin (H&E) stained sections of the gut with light microscopy (Figure 2). At 72 h after WBI, ileal sections showed extensive mucosal damage (Figure 2B). There was severe widespread denudation and altered morphology of the crypts and villi, reparative changes with an increase in cryptogenic activity and abnormal mitotic activity, mucosal necrosis and ulceration. Treatment with rhMFG-E8 resulted in an improvement in histological appearance: there was preservation of villus height and form, and preservation of mucosal layer integrity (Figure 2C). The villus length in the vehicle group was reduced by 42% from the control group, compared to a significantly smaller 19% reduction seen with rhMFG-E8 treatment (Figure 3A). In addition, there was a reduction in the number of nutrient absorbing enterocytes in surviving villi in the vehicle group reflected in an increase in the Goblet cell/enterocyte ratio, which was significantly reduced in the treatment group (Figure 3B). These findings are consistent with the gastrointestinal findings observed in acute radiation syndrome. Based on these and other parameters, we developed a seven point scoring system, the radiation injury intestinal mucosal damage score (RIIMS, range 7–32) to grade the severity of damage (Table 1). The parameters assessed were: goblet cell/enterocyte ratio, villus length as a percentage of normal (sham), villus shape/morphology, crypt cellularity/regeneration, crypt nuclei appearance, Lymph congestion and mucosal necrosis/exfoliation. There was a significant 36% reduction in the RIIMS score in rhMFG-E8 treated rats compared to vehicle treated irradiated animals. rhMFG-E8 treated rats had a score of 18.5±2.4 from a maximum score of 32, compared to vehicle treated rats with a score of 29±2 (Table 2, Figure 3C).

**Table 2:** RIIMS score at 72 h after WBI.

Histological Parameter	Sham	Vehicle	rhMFG-E8
Increase in globlet cell	1	5.75	3.5
Change in villus length	1	5.5	3.25
Villus shape/morphology	1	3.5	2.5
Crypt cellularity/regeneration	1	3.5	2.25
Crypt nuclei appearance	1	3.5	2.25
Lymph congestion	1	3.75	2.5
Mucosal necrosis/exfoliation	1	3.5	2.25
**Total score**	**7**	**29**	**18**

Histological sections of ileum from Sham, Vehicle and rhMFG-E8 treated animals were scored by criteria described in Table 1.

### MFG-E8 gene expression is altered after WBI

**Figure 4:**
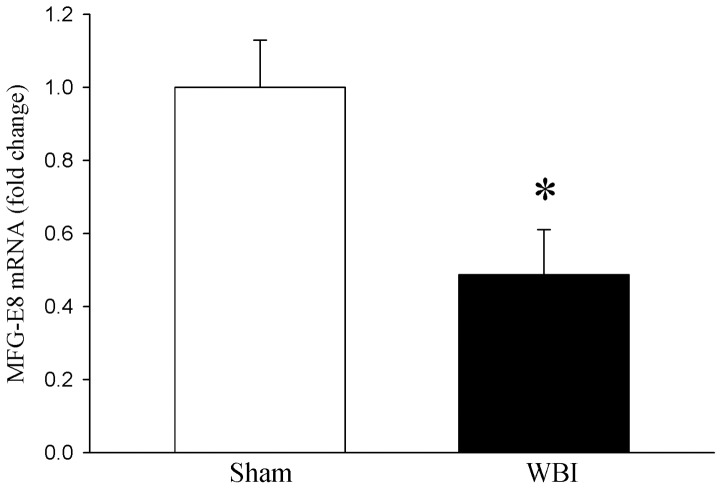
MFG-E8 gene expression in the intestine. Total RNA from gut tissues of Sham and WBI animals were extracted and reverse transcribed. The mRNA expression of MFG-E8 was determined by real-time PCR and fold change over GAPDH is shown. Data are presented as mean ± SE (n = 3–4) and compared by Student's-*t*-test. *P<0.05 vs. Sham.

To examine whether WBI-induced gut injury is associated with alterations of intestinal MFG-E8 gene expression, ileal tissue from sham and WBI-treated animals for 72 h were measured for MFG-E8 gene expression. A significant 51% decrease in MFG-E8 gene expression was observed in WBI-treated animals as compared to sham controls (Figure 4). These data suggest that decrease in intestinal MFG-E8 may contribute to WBI-treated intestinal injury and mortality.

### rhMFG-E8 upregulates p53 expression and increases p21 after WBI

**Figure 5:**
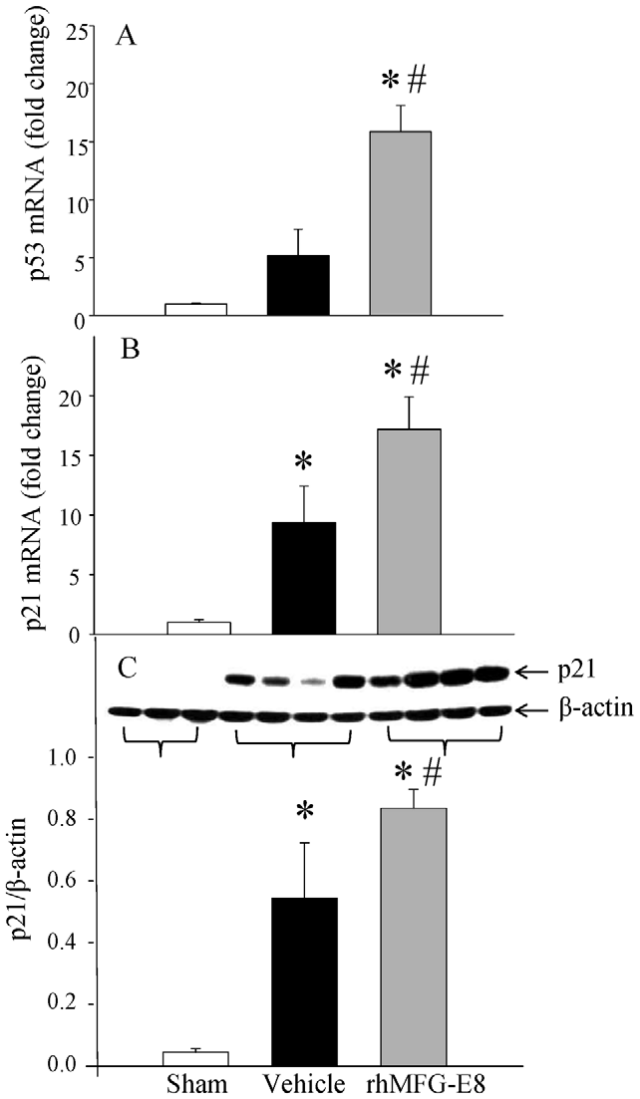
Analysis of cell cycle regulators. Total RNA from gut tissues of Sham, Vehicle and rhMFG-E8 treated animals were extracted and reverse transcribed. The mRNA expression of the cell cycle regulators p53 (A) and p21 (B) were determined by real time PCR and fold change over GAPDH is shown. Proteins were extracted and subjected to Western blotting using p21 and β-actin antibody. A representative blot is shown and the ratio between p21 and β-actin are calculated and plotted (C). Data are presented as mean ± SE (n = 6–8) and compared with Student Neuman Keuls test by ANOVA. * P<0.05 vs. Sham; # P<0.05 vs. Vehicle.

The severity of the GI syndrome is directly correlated to the loss of functional epithelium. Ionizing radiation is a potent cause of apoptosis and several studies have shown an increase in apoptotic activity following GI irradiation. A larger proportion of cell death is however due to catastrophic mitotic activity. It involves cell death occurring either during or shortly after dysregulated mitosis in cells with damaged DNA [16], [17]. The regulatory protein p53, is a tumor suppressor protein which is situated at the crossroads of a network of signaling pathways that are essential for cell growth regulation and apoptosis [18], [19]. As illustrated in Figure 4, analysis of the ileum after WBI showed a 416% increase of p53 expression in vehicle treated animals. Treatment with rhMFG-E8 significantly magnified this response with a 15-fold increase from the control group (Figure 5A). The downstream effector gene p21 increased accordingly with an 8-fold increase in the vehicle group compared to a 16-fold increase after rhMFG-E8 administration (Figure 5B). Similarly, there was a corresponding increase of 13-fold in p21 protein levels in the vehicle group compared to 20-fold increase after rhMFG-E8 treatment.

### rhMFG-E8 increases Bcl-2 in ileal mucosa after WBI

**Figure 6:**
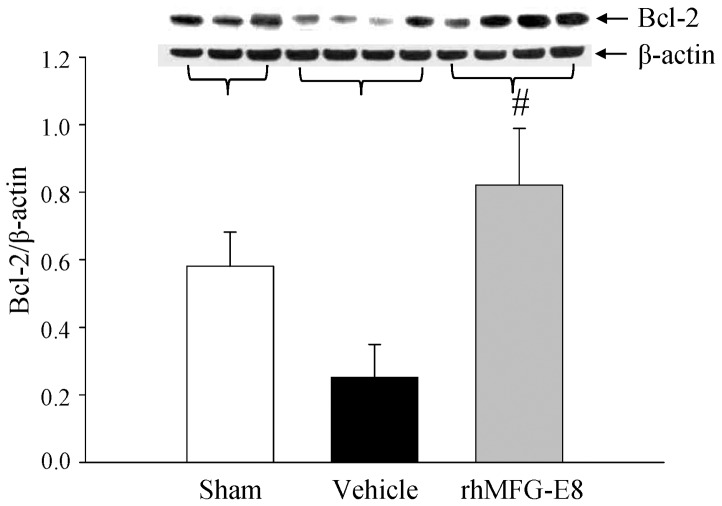
Changes in Bcl-2. (A). Proteins from gut tissues were electrophoresed and Western blotted with bcl-2 and β-actin antibody. A representative blot is shown and the ratio between bcl-2 and β-actin are calculated and plotted. Data are presented as mean ± SE (n = 6–8) and compared with Student Neuman Keuls test by ANOVA. # P<0.05 vs. Vehicle.

To determine the effects of rhMFG-E8 on WBI induced apoptosis, we determined the levels of the anti-apoptotic protein Bcl-2 in the ileum. There was a 43% decrease in Bcl-2 levels in the vehicle group from the sham group as compared to a 41% increase in rhMFG-E8 treated animals (Figure 6). This represented a 141% increase from the vehicle treated animals. The significant difference in the Bcl-2 levels between the vehicle and treatment groups correlate positively with the improved outcomes that we observed in the rhMFG-E8 treated rats.

### rhMFG-E8 protects IEC-6 cells from radiation-induced cell death

**Figure 7:**
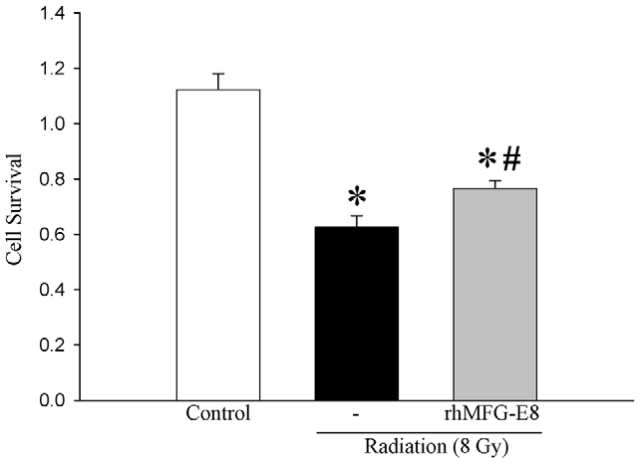
Assessment of cell death. Intestinal epithelial cells (IEC-6) were irradiated at 8 Gy, treated with rhMFG-E8 (0.5 µg/ml) 1 h prior to and immediately after irradiation, and stained with crystal violet. Data are presented as mean ± SE (n = 7) and compared with Student Neuman Keuls test by ANOVA. * P<0.05 vs. Control; # P<0.05 vs. Vehicle.

To determine if intestinal epithelial cells are sensitive to MFG-E8-mediated protection, IEC-6 cells were irradiated at 8 Gy, untreated or pre-treated with 0.5 µg/ml MFG-E8, and stained with crystal violet. Cells treated with 8 Gy showed significant decrease in cell survival as compared to untreated cells. MFG-E8 treatment prior to 1 h followed by immediately after irradiation showed higher staining with crystal violet indicative of protection from cell death (Figure 7). Although indirect, these studies indicated that intestinal epithelial cells could be at least one cell type that MFG-E8 would be able to either protect or possibly restore during radiation in the intestinal tissues.

## Discussion

Ionizing radiation is widely used in medicine and industry. It is utilized in radiotherapy and nuclear imaging for the diagnosis, treatment and monitoring of cancers; in industry for non-destructive testing, in gauges, as radioactive tracers and in the generation of electrical power in nuclear reactors/power plants. Recent world events have highlighted the continuing dangers associated with the utilization of nuclear power. Although nuclear energy is clean and sustainable, it can cause enormous damage in adverse conditions and as illustrated by the Tōhoku earthquake and tsunami and the consequent massive radiation leak at the Fukushima I and other power plants, the risk of radiation exposure cannot be completely eradicated even with the most stringent measures. Ionizing radiation causes various lesions by direct interaction with DNA and indirectly through damage produced by free radicals. After DNA damage has occurred, a number of processes occur in the damaged cell which are important for recovery after radiation exposure but may also play a role in the development of toxicity. Activation of DNA repair, expression of radiation response genes, stimulation of proliferation, and initiation and perpetuation of inflammation could ultimately result in self-perpetuating cascades that lead to vascular damage, tissue hypoxia, widespread cellular dysfunction and death. A probable mechanism for a successful mitigator of radiation injury would be to target these pathways to prevent or reduce toxicity [1], [20]

The amount of damage caused by ionizing radiation is dependent on the dose rate of the radiation and the sensitivity of the organism being irradiated. We therefore determined the sensitivity of our experimental cohort, male Sprague-Dawley rats. We utilized the Gammacell® 1000 Irradiator (Atomic Energy of Canada Ltd) which utilizes Cesium-137 (^137^Cs) as a gamma (γ) rays emitting radiation source. Small amounts of ^137^Cs and ^134^Cs are released into the environment during nearly all nuclear weapon tests and some nuclear accidents, most notably the Chernobyl disaster. It has also been found in the plumes emanating from the continuing leakage at the Fukushima reactors in Japan. A WBI dose of 10 Gy was lethal to 70% of our population of male Sprague-Dawley rats by 21 days (LD_70_/21) (Figure 1A), with the first deaths occurring by Day 7. This corresponds to damage from severe radiation and indicates that the GI syndrome component of ARS was a major contributor to mortality. In this initial study with 10 Gy, we observed 70% mortality within 15 days and the mortality remained the same for an additional week. Therefore, in the treatment study, we only observed the animals for 21 days. Treatment of irradiated rats with rhMFG-E8 (166 µg/kg BW) subcutaneously once a day for 7 days produced significant survival benefits. This was observed as early as day 14 (2-fold increase in survival, p = 0.018) and remained till the end of the observation period by day 21 with a 142% increase in survival (p = 0.027). The survival advantage observed compares favorably even with interventions given in tested animals before irradiation. This was especially remarkable as the survival benefits persisted for over 2 weeks after treatment had been stopped. In addition, a strong correlation exists between body weight changes and survival after WBI.

We used rhMFG-E8 rather than the rodent-derived protein because of its increased translational potential and reduced likelihood of antigenicity in humans. Further, being a recombinant protein, it offers distinct cost advantages for future mass production. We also determined that subcutaneous administration as opposed to an intravenous route would provide a quick route of administration in the event of a nuclear disaster without the need for specialized personnel. Thus, rhMFG-E8 is in a unique niche as a strong candidate for clinical use as a radio-mitigator.

MFG-E8 is a glycoprotein that is comprised of a cleavable signal peptide, followed by two N-terminal EGF-like repeats and two C-terminal Discoidin/F5/8C domains (referred to as F5/8C domains). The arginine-glycine-aspartic acid (RGD) integrin-binding motif on its second EGF domain engages αvβ3/5 integrin heterodimers to facilitate cell adhesion and induce integrin-mediated signal transduction. Each F5/8C domain is composed of an eight-strand anti-parallel β-barrel. Two or three hypervariable loops extend from these and mediate binding to carbohydrate moieties on the surface of cells and in the extracellular matrix. The second C-terminal domain of MFG-E8 also binds to anionic phospholipids of cellular membranes [9], [11], [21]–[23]. MFG-E8 is a potent opsonin for the clearance of apoptotic cells. It is produced by mononuclear cells of immune-competent organs including the spleen and the liver. MFG-E8 facilitates a myriad of inter-cellular interactions, including the maintenance of the intestinal epithelium. Cell proliferation, differentiation, and migration are crucial events required for the maintenance of an intact epithelial layer. Bu *et al* demonstrated that MFG-E8 promotes the migration of intestinal epithelial cells through reorientation of the actin cytoskeleton and that in septic mice, depleting MFG-E8 interrupted enterocyte migration, impaired restitution impeded mucosal healing [12]. MFG-E8 has also been shown to be beneficial in colitis and other forms of intestinal damage [7], [13], [14]. These data indicate that MFG-E8 plays an important role in the maintenance of intestinal epithelial homeostasis and the promotion of mucosal healing, essential attributes in its mitigation of GI impairment after WBI.

The full ARS GI syndrome ensues with acute doses 10 Gy or more, although symptoms may occur as low as 6 Gy. Histological changes include the loss of intestinal crypt cells and breakdown of the mucosal barrier, with sloughing of the epithelial cell layer and denudation of the bowel wall. Impaired barrier function of the gastrointestinal tract results in dehydration, electrolyte imbalance and increased passage of bacteria and their toxins through the intestinal wall into the bloodstream, predisposing to infection and sepsis. Other severe complications include ulceration and necrosis of the bowel wall, leading to stenosis, ileus, and perforation. Recovery is unlikely, as the radiosensitive stem cells in the crypts of the gastrointestinal tract are permanently damaged. Survival is extremely improbable with this syndrome and death usually occurs within 2 weeks. The histology of the small intestine 72 h after WBI (Figure 2) highlights these morphological changes. These changes were attenuated by treatment with rhMFG-E8: histological sections showed conservation of the normal villus structure and increased cryptogenic height and activity pointing to replacement of damaged cells. It is pertinent to also note the paucity of abnormal mitotic nuclei in the crypt after rhMFG-E8 treatment when compared to the Vehicle group. This positive effect of rhMFG-E8 on the gut after WBI irradiation is due, at least in part to its ability to repair damaged intestinal epithelium and preserve gut homeostasis [12].

The ileo-jejunum region of the GI tract has been shown to be particularly sensitive to acute radiation damage. Additionally damage to this portion of the small intestine leads to malabsorption and malnutrition an important systemic effect that worsens morbidity and reduces chances of recovery. Hence the ileum was chosen as a representative segment of the small intestine to study the effects of rhMFG-E8 after WBI [24]. It is not certain that whether WBI-induced intestinal injuries and/or the changes in the representative segment directly influence rat mortality. Nonetheless, our data from the histological sections indicated that MFG-E8 was able to restore the integrity of the ileum.

Hematopoietic parameters such as white blood cell count, red blood cell count, hemoglobin, hematocrit, and platelet count were also assessed at 20 h and one week after WBI. With the exception of the white blood cell count, all measurements were similar to sham levels. The white blood cell count dramatically decreased as early as 20 h and the treatment with MFG-E8 slightly improved the count, but was not significant. Recent unpublished observation from the lab suggests that in animals that survived for 30 days after WBI the white blood cell count returned back to sham levels. Nonetheless, our studies will not exclude the possibility that treatment with rhMFG-E8 in WBI could be protective of the hematopoietic systems. Further studies are warranted for such conclusion.

A further mechanism by which rhMFG-E8 confers a therapeutic advantage after WBI is by upregulating p53. First described in 1979, p53 is a tumor suppressor protein that acts as a regulator of the cell cycle. It is situated at the crossroads of a network of signaling pathways that are essential for cell growth regulation and apoptosis [18], [19], [25]–[28]. In normal unstressed cells, the low levels of p53 protein are maintained as p53 binds to MDM2 and other negative regulators. This promotes its degradation via the ubiquitin/proteasome pathway. After genotoxic stresses, p53 levels accumulate in the cell through the inhibition of its interaction with negative regulators [28]–[30]. Activated p53 binds DNA and activates expression of p21/waf1/cip1 gene which encodes p21, a member of the Cip/Kip family of cyclin-dependent kinase (CDK) inhibitors. The importance of p53 function after irradiation was demonstrated by Kirsch *et al.* [16]. They found that selective deletion of p53 from the GI epithelium sensitized irradiated mice to the GI syndrome and that transgenic mice with overexpression of p53 in all tissues were protected from the GI syndrome after irradiation, a finding corroborated by another study [16], [31]. Treatment with rhMFG-E8 led to an increased expression in the gut of p21 which is known to be critical to cell survival after genotoxic insults [32], [33]. Moreover, Komarova *et al* showed that p21-null animals had accelerated development of lethal GI syndrome after 15 Gy gamma irradiation and suggested that the protective role of p53 in ionizing radiation-induced GI syndrome is mediated by p21 [31]. By increasing the p53 and p21, major regulators of the cell cycle, rhMFG-E8 improves cell survival and protects the genome.

Our results demonstrate that treatment with rhMFG-E8 after WBI upregulates gut Bcl-2. Bcl-2 is an anti-apoptotic protein located on the outer mitochondrial membrane which inhibits caspase activity by preventing the release of cytochrome c from the mitochondria and by binding to the apoptosis-activating factor (APAF-1) [34]–[36]. The increase in Bcl-2 we observed suggests that rhMFG-E8 treatment also acts to prevent apoptotic cell death after WBI. Taken together, our findings reveal that rhMFG-E8 working through various signaling pathways confers a considerable survival advantage when administered several hours after WBI.

We have previously shown that MFG-E8 exerts its beneficial effects in sepsis by increasing apoptotic cell clearance and producing anti-inflammatory properties [37], [38]. It is well recognized that MFG-E8 binds to α_v_β_3_/α_v_β_5_ integrin [39]. Recently, we have elucidated another direct mechanism of MFG-E8 in mediating anti-inflammation. Our study showed that MFG-E8 inhibits LPS-induced TNF-α production via SOCS3 dependent downregulation of NF-κB [40]. However, the precise mechanism of MFG-E8 mediated protection of intestinal tissue after WBI has not been elucidated. One possibility is that MFG-E8-induced p53/p21 upregulation leads to cell cycle arrest in the G1 phase and prevents cells from inappropriately entering into mitosis after WBI. The second scenario is that MFG-E8-mediated p53/p21 upregulation inhibits intestinal tissue apoptosis and thus preserves tissue integrity. In that regard, we showed that Bcl-2, an anti-apoptotic marker is significantly increased in the rat ileum of MFG-E8 treated animals while its expression was diminished in the vehicle group. Future studies are required to pinpoint the exact mechanism in preserving intestinal integrity by MFG-E8 treatment in WBI.

The ongoing possibility of an unexpected nuclear catastrophe necessitates the development of viable mitigators of acute large dose radiation injury. The prevalent cause of death following higher doses of radiation is the GI syndrome component of ARS, which occurs even after rescue by bone marrow replacement. We have demonstrated that rhMFG-E8 given 6 hour after WBI significantly improved survival and ameliorated the GI syndrome. This survival advantage could however involve other body systems. Given its dramatic effect on outcome after WBI, we propose that rhMFG-E8 would likely reduce the long term complications seen after WBI. We have also shown that rhMFG-E8 upregulates p53 and p21 after WBI. While increased p53 has been noted to have different outcomes in various studies, the dramatic improvement in survival with which it is associated in this study points to a unique interaction with rhMFG-E8 to improve cell survival while preserving function, and warrants further investigation [16], [25], [31], 41.
